# For Long-Lived Flies, It's Calorie Quality, Not Quantity, That Matters

**DOI:** 10.1371/journal.pbio.0030237

**Published:** 2005-05-31

In April, the United States Centers for Disease Control and Prevention released a study challenging the conventional wisdom that eating less promotes longevity. The study found that the very thin run roughly the same risk of early death as the overweight. And now the tide seems to be turning against a common explanation for the long-standing observation that restricting food in lab organisms from yeast to mice prolongs life.

Many studies have indicated that it's calorie reduction, rather than the specific source of calories, that increases longevity. That this effect occurs in such diverse organisms suggests a common mechanism may be at work, though none has been definitively characterized. And while calorie restriction enhances longevity in mice, it has not always done so in rats. In a new study, William Mair, Matthew Piper, and Linda Partridge show that flies can live longer without reducing calories but by eating proportionally less yeast, supporting the notion that calorie-restriction-induced longevity may not be as universal as once thought.

Dietary restriction in Drosophila involves diluting the nutrients in the fly's standard lab diet of yeast and sugar to a level known to maximize life span. Since both yeast (which contributes protein and fat) and sugar (carbohydrates) provide the same calories per gram, the authors could adjust nutrient composition without affecting the calorie count, allowing them to separate the effects of calories and nutrients. The standard restricted diet had equivalent amounts of yeast and sugar (65 grams each) and an estimated caloric content of 521, while the yeast-restricted (65 g yeast/150 g sugar) and sugar-restricted (65 g sugar/150 g yeast) diets each had just over 860 calories. The control diet for the flies had equivalent amounts of sugar and yeast (150 grams), amounting to an estimated 1,203 calories. [Fig pbio-0030237-g001]

**Figure pbio-0030237-g001:**
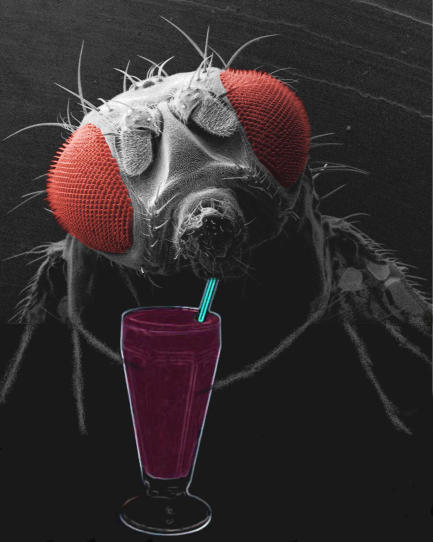
Contrary to popular belief, life span extension by dietary restriction in Drosophila is not explained by calories

First, the authors had to make sure the flies didn't change their eating behavior to make up for a less nutritious diet. (They didn't.) Reducing both nutrients increased the flies’ life spans, but yeast had a much greater effect: reducing yeast from control to dietary restriction levels increased median life span by over 60%.

In a previous study, Mair et al. showed that flies that were switched from dietary-restricted diets to control diets soon began to die at the same rates as flies accustomed to the control diet. In this study, the authors studied the effects of switching yeast and sugar. Forty-eight hours after being switched from normal diets to yeast-restricted diets, flies were no more likely to die than flies fed the yeast-restricted diet from the beginning. In contrast, those switched from the standard restriction diet to the sugar-restricted diet began to die at the same rate as flies on the control diet.

The authors also ruled out the possibility that bacteria—attracted to high-nutrient food—might be influencing fly survival. Altogether these results make a strong case that calories per se are not the salient factor in prolonging life—at least in fruitflies. The dramatic impact of reducing yeast suggests that protein or fat plays a greater role in fly longevity than sugar. This in turn suggests, the authors argue, that yeast and sugar trigger different metabolic pathways with different effects on life span.

Why might different factors promote longevity in flies and rats? It could be that the caloric-restriction/longevity paradigm needs more rigorous review—though a vast body of literature does support it. Or it may be that the animals use the same strategy for dealing with food shortages—shifting resources from reproduction to survival, for example—but have evolved different mechanisms for doing so that reflect each species's life history, diet, and environment. Whatever explains the disparity, this study should give researchers interested in caloric restriction plenty to chew on.

